# Subcutaneous dirofilariasis

**DOI:** 10.1093/omcr/omae192

**Published:** 2025-02-22

**Authors:** Poorvi Sharma, Nitin Gupta, Idzi Potters, Anjely Sebastian, Muralidhar Varma, Juhi Irfana Basheer, R Balakrishnan, Nancy Namrata Mahapatra, Vishwapriya M Godkhindi, Kanthilatha Pai, Anuradha Calicut Kini Rao

**Affiliations:** Department of Ear, Nose and Throat, Kasturba Medical College, Manipal, Manipal Academy of Higher Education, Madhavnagar, Manipal 576104, Karnataka, India; Department of Infectious Diseases, Kasturba Medical College, Manipal, Manipal Academy of Higher Education, Madhavnagar, Manipal 576104, Karnataka, India; Department of Clinical Sciences, Institute of Tropical Medicine, Nationalestraat, Antwerp 2000, Belgium; Department of Infectious Diseases, Kasturba Medical College, Manipal, Manipal Academy of Higher Education, Madhavnagar, Manipal 576104, Karnataka, India; Department of Infectious Diseases, Kasturba Medical College, Manipal, Manipal Academy of Higher Education, Madhavnagar, Manipal 576104, Karnataka, India; Department of Ear, Nose and Throat, Kasturba Medical College, Manipal, Manipal Academy of Higher Education, Madhavnagar, Manipal 576104, Karnataka, India; Department of Ear, Nose and Throat, Kasturba Medical College, Manipal, Manipal Academy of Higher Education, Madhavnagar, Manipal 576104, Karnataka, India; Department of Pathology, Kasturba Medical College, Madhavnagar, Manipal, Manipal Academy of Higher Education, Manipal 576104, India; Department of Pathology, Kasturba Medical College, Madhavnagar, Manipal, Manipal Academy of Higher Education, Manipal 576104, India; Department of Pathology, Kasturba Medical College, Madhavnagar, Manipal, Manipal Academy of Higher Education, Manipal 576104, India; Department of Pathology, Kasturba Medical College, Madhavnagar, Manipal, Manipal Academy of Higher Education, Manipal 576104, India

**Keywords:** Infectious diseases and tropical medicine

## Clinical image

**Figure 1:**
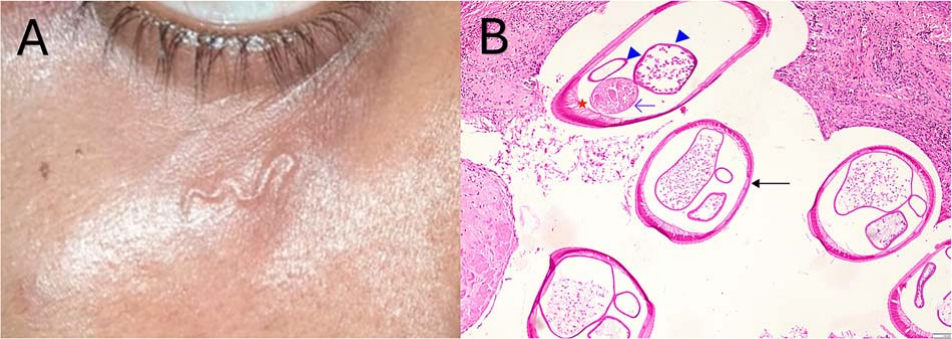
Subcutaneous worm below the right eye (A); Haematoxylin and eosin stain (200X magnification) of the cross-section of the worm shows smooth longitudinally multilayered ridged cuticle *sans* bumps (thick arrow), internal lateral ridge with tall coelomyrian musculature (asterisk), paired reproductive tubules (arrowhead) and intestine (thin arrow) (B).

A 19-year-old male student, resident of Udupi, Karnataka, India, without any co-morbidities or history of travel, presented to a primary care physician with acute onset wiggling sensation below the right eye. This was accompanied by a subcutaneous structure resembling a worm (Fig. 1A). There was no fever, itching, pain or redness. The patient was managed conservatively with anti-inflammatory medications in the hope of a spontaneous resolution. Over a few days, a swelling developed at the site, which grew in size over three months.

The patient presented to our tertiary care centre with a subcutaneous nodule beneath the right eye. This nodule was excised, and a worm was noted on gross examination. On histopathological examination, longitudinal ridges on a thin cuticle and coelomyarian musculature were observed (Fig. 1B), leading to the morphological identification of possible *Dirofilaria* repens or *Dirofilaria* spp Hong Kong genotype. No hypereosinophilia or microfilaremia was noted on the peripheral blood smear. No anti-parasitic medications were prescribed, and the patient remained asymptomatic on follow-up three weeks later.

Dirofilariasis is caused by a zoonotic parasite accidentally introduced into humans when an infected mosquito takes a blood meal [1]. It presents with either pulmonary involvement (*Dirofilaria immitis*) or as subcutaneous dirofilariasis (*D. repens*, *D. tenuis*, *Dirofilaria* spp. Hong Kong genotype) [2]. Subcutaneous dirofilariasis is reported in several regions of Asia, Africa, and Europe. After the third stage larva matures into adult worms, it may migrate to multiple places, leading to transient swellings, eventually forming a nodule in the eyes, peri-orbital region or other upper body parts. In human infections, a single worm is usually involved, probably due to the host’s immune system decreasing the chances of maturation of multiple worms. Consequently, microfilaremia is rare due to the lack of reproduction inside the human host [1, 3]. A gross examination of adult *Dirofilaria* spp shows a worm of 300–400 μm diameter and a length of around 10 cm [4]. Histopathological examination can help with the speciation, as longitudinal ridges on the cuticle are absent in *Dirofilaria immitis* [4]. Surgical excision is the preferred treatment. Dirofilariasis can be prevented in endemic areas by avoiding mosquito bites (minimising exposed body surface area, using insect repellents, and using insecticide-treated bed nets).

Dirofilariasis should be suspected in individuals with worm-like swelling or subcutaneous nodules, especially when they have a residence history or travel to endemic areas in Asia, Africa and Europe.
